# Molecular detection of *Hepatozoon* spp. and *Cytauxzoon* sp. in domestic and stray cats from Madrid, Spain

**DOI:** 10.1186/s13071-017-2056-1

**Published:** 2017-03-13

**Authors:** David Díaz-Regañón, Alejandra Villaescusa, Tania Ayllón, Fernando Rodríguez-Franco, Gad Baneth, Lydia Calleja-Bueno, Mercedes García-Sancho, Beatriz Agulla, Ángel Sainz

**Affiliations:** 1 Department of Animal Medicine and Surgery, College of Veterinary Medicine, Complutense University of Madrid, Avda. Puerta de Hierro s/n, 28040 Madrid, Spain; 2 Instituto Nacional de Infectologia Evandro Chagas, Fiocruz, Avenida Brazil 4365, CEP 21040-900 Rio de Janeiro, Brazil; 3 Koret School of Veterinary Medicine, Hebrew University of Jerusalem, P.O. Box 12, 76100 Rehovot, Israel

**Keywords:** Cat, Central Spain, *Cytauxzoon* sp., *Hepatozoon canis*, *Hepatozoon felis*, PCR

## Abstract

**Background:**

Different species of apicomplexan protozoans of the genera *Hepatozoon* and *Cytauxzoon* can infect domestic cats, but their epidemiology and clinical relevance are not fully understood. The aim of this study was to assess the molecular prevalence of *Hepatozoon* spp. and *Cytauxzoon* spp. and to identify associated risk factors and clinical and laboratory abnormalities in a population of cats from Madrid, Spain.

**Methods:**

Six hundred and forty-four client-owned and stray cats from Madrid, Spain, were included in this study. DNA samples were analyzed by two polymerase chain reaction (PCR) tests to detect a partial sequence of the 18S rRNA gene of *Hepatozoon* spp. and *Cytauxzoon* spp. In order to evaluate possible associations between infection by these protozoans and epidemiological or clinical parameters, data were collected related to: the season of sample collection, age, gender, spayed/neutered status, breed, living area, lifestyle, outdoor access, contact with other animals, prey on wild animals, history of tick or flea infestation, travel history, ectoparasiticide treatment, previous blood transfusion, previous tetracycline administration in the last 60 days, Feline Leukemia virus (FeLV) and Feline Immunodeficiency virus (FIV) status, positivity to other vector-borne diseases, the presence or absence of clinical signs and hematological or biochemical alterations.

**Results:**

DNA of *Hepatozoon* spp. and *Cytauxzoon* sp. was amplified from the blood of 10 (1.6%) and 8 (1.2%) cats, respectively. Previous treatment with tetracyclines in the last 60 days, previous administration of blood transfusion, a decrease in haematocrit and an increase in creatinine were associated with *Hepatozoon* spp. infection*. Cytauxzoon* sp. infection was more frequent in samples collected during the winter months and in cats living in rural areas. This infection was associated with a FIV-positive status. Some of the cats that were positive for *Hepatozoon* spp. or *Cytauxzoon* sp. had been exposed to other vector-borne pathogens, such as *Ehrlichia canis* and *Bartonella henselae*.

**Conclusions:**

Our results indicate that cats from Madrid, central Spain, are infected with *Hepatozoon* spp. and *Cytauxzoon* sp., although with a low prevalence. Further studies are needed to determine the virulence of these agents in Spanish cats.

## Background

Feline vector-borne diseases are considered emerging or re-emerging diseases. Their epidemiology is changing, showing a wider geographical distribution and increasing global prevalence, due to climate changes and environmental, demographic and human behavioural factors [1–3]. All these factors favour contact between wildlife reservoir hosts and invertebrate vectors with humans and pets. Although several vector-borne agents cause disease and even death in the domestic cat, the detection of others can be challenging because of their occurrence in healthy cats, the presence of unspecific clinical signs in infected animals or the simultaneous occurrence with other infections [4]. In fact, the importance of some of these pathogens as a cause of disease has not been clearly determined [5].

The genus *Hepatozoon* comprises more than 340 species of apicomplexan protozoans of the family Hepatozoidae, are closely related to piroplasms and haemosporids and infect a wide variety of amphibians, reptiles, birds, and mammals [6]. The main transmission route is through the ingestion of the definitive host, a hematophagous arthropod, by the intermediate vertebrate host [6]. *Hepatozoon* infection is well recognized in dogs, in which two species are described, *Hepatozoon canis* and *Hepatozoon americanum* [7]. However, in cats infection is poorly understood [8]. Hepatozoonosis of the domestic cat has been reported from different countries, including Brazil [9, 10], France [11], India [12], Israel [8, 13], Nigeria [14], Portugal [4, 15], South-Africa [16], Spain [17–19] and the USA [20]. Feline hepatozoonosis is associated with infection of muscle tissues. It has been generally considered that the infection is mostly subclinical, although pathogenic effects may be exacerbated in stressed, immunocompromised animals or in concomitant infections [8].

On the contrary, infection in domestic cats with *Cytauxzoon felis* is described as a cause of a progressive usually fatal disease. *Cytauxzoon* spp. are apicomplexan haemoparasites belonging to family Theileriidae transmitted by ticks which infect wild and domestic felids [21]. Several *Cytauxzoon* spp. have been identified with *C. felis* being the most important. Historically, infection with *Cytauxzoon* spp. in domestic cats was only reported in North and South America, but it has been more recently described in felids from Europe [22–26].

Limited previous molecular surveys have described infection with *Hepatozoon* spp. [18, 19, 22, 26] and *Cytauxzoon* spp. [22] in domestic cats from Spain. Therefore, the main objectives of the current study were to identify the presence and determine the prevalence of vector-borne agents of the genera *Hepatozoon* and *Cytauxzoon* in client-owned and stray cats from Madrid, central Spain; and, due to the scarce information available on the epidemiology and clinical manifestations of feline hepatozoonosis and cytauxzoonosis in European countries, to identify risk factors and clinical signs associated with these infections in cats.

## Methods

### Animals and samples and data collection

Samples were collected from 644 cats between September 2005 and December 2008. The cats attended the Veterinary Teaching Hospital (VTH) of the Complutense University of Madrid, three different private veterinary clinics, or five animal protection societies in the Madrid area. These animals were mostly enrolled in a previous study that assessed exposure and infection with *Ehrlichia* spp*.*, *Anaplasma* spp*.*, *Neorickettsia* spp*.*, *Leishmania* spp*.*, and *Bartonella* spp., in client-owned and stray cats [5].

Written consent for patient enrolment was obtained for every cat from owners or animal protection societies. No specific inclusion or exclusion criteria were established; therefore, any cat that attended the VTH or private clinics from which blood samples were collected for routine laboratory testing was enrolled in the study. Stray cats at the animal protection societies were randomly selected from those that were neutered/spayed.

Whenever available, different epidemiological and clinical data were obtained from each cat. Feline Leukemia virus (FeLV) and Feline Immunodeficiency virus (FIV) status were determined by the use of a commercial kit (IDEXX Laboratories, Westbrook, Maine, USA). Cats were classified as healthy or unhealthy depending on the clinical history and presence of clinical signs (general signs, digestive signs, cardiovascular/respiratory signs, ocular signs, musculoskeletal abnormalities, renal signs, and neurological signs). In some cases, especially in cats from shelters, the collected data were scarce and consequently in some variables the sample size does not include all the 644 studied cases. Nonetheless, all the available data have been included due to the lack of studies regarding this population and with the aim of evaluating cats from different sources.

### PCR amplification and sequencing

As described by Ayllón et al. [5], DNA was extracted from 200 μl whole blood samples using an UltraCleanTM DNA Blood Spin Kit (Mo Bio Laboratories, Carlsbad, USA) following the manufacturer’s instructions. All DNA samples used for PCR were stored at -80 °C until use. DNA was then quantified by spectrophotometry and assessed for quality at 260/280 nm and 260/230 nm by spectrophotometry (NanoDrop^TM^; Thermo Scientific, Waltham, USA).

Subsequently, two previously described PCR protocols based on piroplasmid 18S rRNA gene were performed on the 644 samples for detection of *Hepatozoon* spp. and *Cytauxzoon* spp. DNA [19, 27] with some modifications. Table 1 shows the primers and protocols employed in the PCR assays. Both PCR reactions were carried out in a 25.5 μl reaction mixture containing 5 μl of genomic DNA, 12.5 μl Premix Ex Taq™ (Lab. Conda, Madrid, Spain) and 0.25 μl of each corresponding primer (50 μM). Negative and positive controls were included with each run.

**Table 1:** Primers and protocols used for the amplification of *Cytauxzoon* sp., *Hepatozoon* spp. and housekeeping GAPDH gene control

	PCR primers (5′-3′)	PCR conditions	Product size (bp)
PCR-1	F: CCAGCAGCCGCGGTAATTC	94 °C, 3 min; 35 cycles [94 °C, 30 s, 64 °C, 45 s, 72 °C, 30 s]; 72 °C, 7 min	373
	R: CTTTCGCAGTAGTTYGTCTTTAACAAATCT		
PCR-2	F: CCTGGTTGATCCTGCCAG	96 °C, 3 min; 40 cycles [96 °C, 1 min, 65 °C, 1 min, 72 °C, 2 min]; 72 °C, 1 min	1,675
	R: CGACTTCTCCTTCCTTTAAG		
Housekeeping (GAPDH)	F: CCTTCATTGACCTCAACTACAT	95 °C, 1 min; 45 cycles [94 °C, 4 s, 57 °C, 4 s, 72 °C, 3 s]; 72 °C, 1 min	282
	R: CCAAAGTTGTCATGGATGACC		

The reactions were performed in an automatic DNA thermal cycler MasterCycler® ep Gradient (Eppendorf, Hamburg, Germany). The products of amplification reactions were visualized by electrophoresis on 1.5% agarose gel (PCR-1) and 1% (PCR-2) containing ethidium bromide (10 mg/ml) run at 115 V for 30 min for the first PCR and 90 V for 30 min for the second PCR.

Negative samples were submitted to the housekeeping gene glyceraldehyde-3-phosphate dehydrogenase (GAPDH) amplification to assess the extraction quality (DNA presence and absence of inhibition factors) in a single PCR round as described by Birkenheuer et al. [28]. Primers and protocol employed in this PCR assay are shown in Table 1.

The reaction products were purified using SpeedTools PCR Clean-Up kit (Biotools, Madrid, Spain). Purified amplified DNA fragments from the positive samples were subjected to sequencing in an automated sequencer 3730 DNA Analyzer by using the Big Dye Terminator version 3.1(Applied Biosystems) by Secugen S.L (Madrid, Spain). Comparisons with sequences deposited in GenBank were made using the Basic Local Alignment Search Tool (BLAST).

### Statistical analysis

Statistical analysis of the results was performed by the “Departamento de Ayuda a la Investigación, Área de Informática y Comunicaciones” of the Complutense University of Madrid, using the commercially available statistical software SAS, version 9.4 (SAS Institute, Cary, NC, USA). Statistical associations between PCR results and haematological, biochemical and epidemiological data were obtained from each cat and analysed by Chi-square test or Fisher’s exact test and odds ratio (OR). The significance level was set at *P* < 0.05.

## Results

### Cat description

Among the 644 cats included in this study, 506 (78.6%) were client-owned and 138 (21.4%) were stray cats. Data collected regarding epidemiological information are shown in Tables 2 and 3. It was not possible to obtain these data from all the animals included in the study, particularly in the case of stray cats.

**Table 2:** Comparison of prevalence of *Hepatozoon* spp. and *Cytauxzoon* sp. in association with different epidemiological data

	Total no. of cats (%)	No. of positive cats (%)	
		*Hepatozoon* spp.	*Cytauxzoon* sp.
	644	10 (1.6)	8 (1.2)
Season of sample collection	644		
Spring	201 (31.2)	4 (2.0)	1 (0.5)
Summer	88 (13.7)	0	0
Autumn	168 (26.1)	1 (0.6)	1 (0.6)
Winter	187 (29.0)	5 (2.7)	6 (3.2)*
Age	547		
Kitten (< 1 year-old)	107 (19.6)	2 (1.9)	1 (0.9)
Adult (1–10 year-old)	315 (57.6)	2 (0.6)	3 (1.0)
Older cat (> 10 year-old)	125 (22.8)	0	0
Gender	589		
Male	277 (47.0)	2 (0.7)	2 (0.7)
Female	312 (53.0)	3 (1.0)	2 (0.6)
Spayed/Neutered	548		
Yes	311 (56.7)	3 (1.0)	1 (0.3)
No	237 (43.3)	2 (0.8)	3 (1.3)
Breed	585		
European shorthair	439 (75.0)	4 (0.9)	3 (0.7)
Non-European shorthair	146 (25.0)	1 (0.7)	1 (0.7)
Living area	440		
Urban	248 (56.4)	3 (1.2)	0
Periurban	117 (26.6)	1 (0.9)	1 (0.9)
Rural	75 (17.0)	0	2 (2.7)*
Lifestyle	644		
Client-owned	506 (78.6)	6 (1.2)	4 (0.8)
Stray	138 (21.4)	4 (2.9)	4 (2.9)
Outdoor access	506		
Yes	269 (53.2)	4 (1.5)	6 (2.2)
No	237 (46.8)	2 (0.8)	1 (0.4)
Contact with other animals	514		
Yes	392 (76.3)	8 (2.0)	7 (1.8)
No	122 (23.7)	0	0
Prey on wild animals	415		
Yes	157 (37.8)	1 (0.6)	2 (1.3)
No	258 (62.2)	3 (1.2)	1 (0.4)
Previous tick infestation	413		
Yes	30 (7.3)	1 (3.3)	0
No	383 (97.7)	3 (0.8)	3 (0.8)
Previous flea infestation	415		
Yes	64 (15.4)	0	0
No	351 (84.6)	4 (1.1)	3 (0.9)
Travel history	372		
Yes	141 (38.0)	0	1 (0.7)
No	231 (62.0)	3 (1.3)	2 (0.9)
Ectoparasiticide treatment	488		
Yes	103 (21.1)	2 (1.9)	1 (1.0)
No	385 (78.9)	5 (1.3)	6 (1.6)
Previous blood transfusion	389		
Yes	5 (1.3)	1 (20.0)*	0
No	384 (98.7)	2 (0.5)	3 (0.8)
Tetracyclines treatment	391		
Yes	13 (3.3)	2 (15.4)*	0
No	378 (96.7)	1 (0.3)	3 (0.8)
FeLV	482		
Yes	35 (7.3)	2 (5.7)	0
No	447 (92.7)	5 (1.1)	7 (1.6)
FIV	484		
Yes	26 (5.4)	0	2 (7.7)*
No	458 (94.6)	7 (1.5)	5 (1.1)

*P < 0.05 (statistically significant differences)

**Table 3:** Clinical parameters and relevant alterations in laboratory findings of the cats studied

	Total no. of cats (%)	No. of positive cats (%)	
		*Hepatozoon* spp.	*Cytauxzoon* sp.
	644	10 (1.6)	8 (1.2)
Clinical signs	542		
Yes	380 (70.1)	3 (0.8)	3 (0.8)
No	162 (29.9)	2 (1.2)	1 (0.6)
Haematology			
Haematocrit	463		
High	28 (6.0)	1 (3.6)	0
Low	87 (18.8)	3 (3.5)	1 (1.2)
Normal	348 (75.2)	2 (0.5)*	3 (0.9)
Leukocytes	460		
High	88 (19.1)	2 (2.3)	1 (1.1)
Low	63 (13.7)	1 (1.6)	0
Normal	309 (67.2)	3 (1.0)	3 (1.0)
Platelets	168		
High	14 (8.3)	0	0
Low	89 (53)	0	1 (1.1)
Normal	65 (38.7)	3 (4.6)	0
Biochemistry			
Glucose	378		
High	138 (36.5)	0	1 (0.7)
Low	3 (0.8)	0	0
Normal	237 (62.7)	3 (1.3)	3 (1.3)
Urea	419		
High	101 (24.1)	1 (1.0)	0
Low	74 (17.7)	1 (1.4)	0
Normal	244 (58.2)	2 (0.8)	4 (1.6)
Creatinine	859		
High	82 (18.6)	3 (3.7)*	0
Low	16 (3.6)	0	0
Normal	342 (77.7)	2 (0.6)	4 (1.2)
Total protein	408		
High	135 (33.1)	1 (0.7)	1 (0.7)
Low	35 (8.6)	1 (2.9)	0
Normal	238 (58.3)	2 (0.8)	3 (1.3)
Alanine transaminase	441		
High	76 (17.2)	2 (2.6)	2 (2.6)
Normal	365 (82.8)	3 (0.8)	1 (0.3)

*P < 0.05 (statistically significant differences)

Clinical signs of disease and/or laboratory abnormalities were found in 380 out of 542 (70.1%) cats for which these data were available. General clinical signs were recorded in 33% (179/542); 31.2% (169/542) had gastrointestinal signs; 23.3% (126/542) had cardiovascular/respiratory signs; 22.7% (123/542) had renal signs; 9.6% (52/542) had ocular signs; 7.6% (41/542) had musculoskeletal abnormalities; and 3.2% (17/525) had neurological signs.

### PCR amplification and sequencing

Of the 644 sampled cats, *Hepatozoon* spp. DNA was amplified from 10 animals (1.6%). Sequencing confirmed *H. felis* infection in 9 cats (99–100% identity to GenBank KC138534.1 in 8 cats and 100% to HQ020489.1 and 99% to KC138534.1 in 1 cat). *Hepatozoon canis* infection was confirmed in another cat, with 99% identity to different close GenBank sequences, including isolates from ticks from Palestine (KT587790.1) and Pakistan (JX441117.1), from dogs from Israel (KC138535.1), India (JN584477.1) and Spain (AY461378.2), from wild canids from Israel (KJ868815.1), India (HQ829447.1) and Spain (AY731062.1) and from cats from Israel (KC138532.2 and KC138531.2).

*Cytauxzoon* sp. DNA was amplified in 8 of the cat samples (1.2%). The sequenced products showed 99–100% identity with 18S rRNA gene of *Cytauxzoon* sp. cat/France 1/2008 (EU622908.1), *Cytauxzoon* sp. wild cat isolate 03/04326 (KT223486.1) and *Cytauxzoon manul* (KU710344.1). No coinfection of *Hepatozoon* spp. and *Cytauxzoon* sp. was detected in the cat population of the study.

### Evaluation of epidemiological and clinical data

Results for the evaluation of associations between positivity by PCR to *Hepatozoon* spp. and *Cytauxzoon* sp. and the epidemiological and clinical data collected for the cat population of this study are shown in Tables 2 and 3. When analyzing these epidemiological data, age, gender, being spayed/neutered and the breed of the cat did not show any statistically significant association with these infections.

*Cytauxzoon* sp. infection was detected mostly (6/8) in blood samples drawn during winter months and infection was associated with the winter in comparison with the other seasons together (*P =* 0.0091, OR = 7.54, 95% CI: 1.50–37.71). The other two positive cats for this agent included in the study were sampled during spring and autumn. The season when the cats were included in the study did not have a statistically significant influence on *Hepatozoon* spp. prevalence (*P* = 0.2774), although five and four out of the 10 positive samples were drawn in winter and spring, respectively. Cats living in rural areas had a higher prevalence of *Cytauxzoon* sp. infection (2.7%, 2/73) compared to cats living in periurban (0.9%, 1/116) or urban (0/248) areas (*P* = 0.0463).

There was no statistically significant association between positivity by PCR to *Hepatozoon* spp. or *Cytauxzoon* sp. and being a client-owned or a stray cat (*P* = 0.1492 and *P* = 0.0.691, respectively) or having outdoor access (*P* = 0.6894 and *P* = 0.1277, respectively). However, when positive results to *Hepatozoon* spp. and to *Cytauxzoon* sp. were considered as a whole, stray cats were more likely to be PCR positive (*P* = 0.0348), with a higher prevalence (5.8%, 8/138) than client-owned cats (2%, 10/506).

Previous history of tick or flea infestation, prey on wild animals, lack of ectoparasiticide treatment and travel history were not associated with positivity for these pathogens. However, previous treatment with tetracyclines in the last 60 days and previous administration of blood transfusion were associated with *Hepatozoon* spp. infection (*P* = 0.0030, OR = 68.54, 95% CI: 5.77–813.77; and *P* = 0.0382, OR = 47.75, 95% CI: 3.56–639.51, respectively), but not with *Cytauxzoon* sp. infection.

When assessing FeLV/FIV status of the cats positive for *Hepatozoon* spp. or *Cytauxzoon* sp. infections, although in a non-significant manner (*P* = 0.0854, OR = 5.35, 95% CI: 1.00–28.67), a higher trend was found of *Hepatozoon* spp. infection in cats positive for FeLV (5.7%, 2/35) in comparison with the cats negative for FeLV (1.1%, 5/447). All the cats with amplification of *Hepatozoon* spp. DNA were FIV-negative. On the contrary, none of the cats with amplification of *Cytauxzoon* sp. DNA was positive for FeLV, but two of them were positive for FIV (7.7%, 2/24), while the prevalence of *Cytauxzoon* sp. infection in FIV-negative cats was 1.1% (5/453). Consequently, FIV infection was found statistically associated with *Cytauxzoon* sp. infection (*P* = 0.0494, OR = 7.55, 95% CI: 1.39–40.93).

There was no statistically significant association between the pathogens studied and the presence or absence of any clinical signs. However, *Hepatozoon* spp. infection was associated with a decrease in haematocrit (*P* = 0.0375) and an increase in creatinine (*P* = 0.047).

Based on immunofluorescent antibody (IFA) tests, some of the cats with *Hepatozoon* spp. or *Cytauxzoon* sp. infection had been exposed to other vector-borne pathogens. Specifically, one cat with *Hepatozoon* spp. infection and another with *Cytauxzoon* sp. infection were seropositive for *Ehrlichia canis*. In addition, four of the *Hepatozoon* spp.-infected cats and other four *Cytauxzoon* sp.-infected cats were serologically positive for *Bartonella henselae*. It was possible to confirm this concurrent infection with *Bartonella* spp. by PCR from blood samples in one *Cytauxzoon* sp.-positive stray cat, resulting in a statistically significant association between these infections (*P* = 0.0247).

## Discussion

The detection of *Hepatozoon felis*, *H. canis* and *Cytauxzoon* sp. in blood samples from client-owned and stray cats from Madrid reported in the present study suggests that the feline population of this region is infected with these apicomplexans, although with a low prevalence.

The diagnosis of *Hepatozoon* spp. in cats from central Spain reported herein support that these protozoans are widespread in the Iberian Peninsula, taking into account the previous descriptions in cat populations from Portugal and different regions of Spain [4, 15, 17–19]. The sequencing of the PCR products amplified from the cat samples in this study showed that both *H. felis* and *H. canis* can infect cats in Spain, although *H. felis* is by far more common, with only one cat infected with *H. canis*. A similar higher ratio of *H. felis versus H. canis* infection in domestic cats was also found in a study from Israel where only two cats out of 55 *Hepatozoon*-spp.-positive cats were detected with *H. canis* infection and 53 cats out of a total of 153 tested were positive for *H. felis* [13]. The *Hepatozoon* infection prevalences described in previous Spanish studies varied from 0.6% in one study [17], 4% in cats from the Barcelona area [19] and 16% in a cat colony from Barcelona [18], compared to the prevalence in our study of 1.6%. These differences in prevalence rates can be due to the vector distribution, characteristics of the cat populations in each case or differences in the PCR technique and type of sample employed. No cats were positive for *Hepatozoon silvestris*, a newly described feline *Hepatozoon* species found in the European wildcats (*Felis silvestris silvestris*) in Bosnia-Herzegovina [29]. *Hepatozoon* spp. are transmitted by ingestion of the final host containing mature oocysts by the intermediate host [6]. Although the vectors of feline hepatozoonosis are still unknown, it is expected that *H. felis* would be transmitted by an hematophagus arthropod, as demonstrated for other *Hepatozoon* spp. transmited by fleas, ticks, mites, lice, mosquitoes and sand flies [30, 31]. Variations in the possible vector distribution can explain, at least partially, the differences in *Hepatozoon* spp. infection rates described in studies performed in different regions. In our study, no statistical association was found between positivity to *Hepatozoon* spp. and a previous history of tick or flea infestation.

Other transmission routes have been described for some *Hepatozoon* spp., including intrauterine transmission and carnivorism [13, 32, 33]. It was possible to collect information regarding prey on wild animals from four out of the ten *Hepatozoon* spp.-positive cats in the current study and only one of them was reported to ingest rodents, birds and other wild animals. However, the other three *Hepatozoon* spp*.*-positive animals were stray cats whose hunting activity was unknown.

No association was found between the lifestyle of the cats (client-owned or stray) and positivity to *Hepatozoon* spp. However, a higher prevalence of *Hepatozoon* spp. infection has been described in cats from a colony in the Barcelona area (16%) [18] and in cats with outdoor access in comparison with strictly indoor cats [4, 13]. In our study, positivity to *Hepatozoon* spp. was not statistically related to the outdoor access, but this information was available only for six out of the ten cats positive for *Hepatozoon* spp. infection and four of them had outdoor access.

One interesting finding of the current study is the absence of older cats (more than ten year-old) among *Hepatozoon* spp.-infected cats. In agreement, a previous retrospective study found that four out of seven cats with hepatozoonosis were two year-old or younger [8]. In a later study, transplacental transmission of *H. felis* was suggested as a mode of transmission as *H. felis* DNA was shown to be present in foetuses of *H. felis*-positive queens [13]. However, Maia et al. [4] found that the prevalence of *Hepatozoon* spp. was higher in cats older than 60 months (5 years).

In addition, five of the *Hepatozoon* spp.-positive cats included in the current study were sampled during the winter months (January and February), four during spring (April and May) and one in autumn (October). Due to the probable vector-borne transmission of *H. felis*, this finding could be related to changes in vector activity in different seasons of the year. The finding of a higher prevalence in winter is unexpected, as the abundance of competent vectors could decrease during the colder months of the year. However, Baneth et al. [8] described the detection of feline hepatozoonosis in the Tel-Aviv area mainly in winter, suggesting that it could be due to the occurrence of the infection during these months, following a long life-cycle of the protozoa in which gamonts appear in the peripheral blood several months after the infection takes place in the warmer months, or due to a chronic infection, with no direct relation to the time of initial infection.

Feline hepatozoonosis is poorly understood [8]. It is generally considered that *Hepatozoon* spp. infection in cats has a low virulence, being mostly subclinical [13, 18], but there is scant clinical information on the disease. *Hepatozoon felis* usually infects the myocardial and skeletal muscles, with no significant local inflammatory response [13, 34]. In the present study, no association was found between the presence or absence of clinical signs and *Hepatozoon* spp. infection. However, the presence of lower haematocrit values (< 24%) and higher creatinine levels (>1.8 mg/dl) were statistically associated with this infection. When analysing this result, it should be taken into account that two of the three cats with *Hepatozoon* spp. infection that have low haematocrit and high alanine-amino transaminase (ALT) value, were also FeLV positive, with one of them also being seropositive for *B. henselae*. In addition, clinical signs and/or haematological alterations found in the cats of the study should be interpreted cautiously, due to possible coinfections with other pathogens not tested in the current study, such as haemotropic mycoplasmas. The presence of coinfections with FeLV or FIV and other vector-borne pathogens has been frequently described in feline hepatozoonosis [4, 8, 13, 15, 18, 19] and complicate the interpretation of clinical and laboratory findings. In this sense, it has been suggested that hepatozoonosis may develop in association with immunosupression induced by a concurrent disease that can lead to the intensification of parasitemia [8]. In our study, a statistically significant association was found between *Hepatozoon* spp.-positivity and previous tetracycline treatment or previous blood transfusion. When analysing these results, it should be considered that the total animals included in this study with history of treatment with these drugs in the last 60 days (3.3%, 13/391) or that received blood transfusion previously (1.3%, 5/389) is low. Tetracycline drugs are frequently prescribed when a suspicion of a vector-borne disease exists, however, it has not been described to be effective against *Hepatozoon* spp. Furthermore, the *Hepatozoon* spp.-positive cat that had received a blood transfusion was also treated with doxycycline and was FeLV and *B. henselae*-positive.

*Cytauxzoon* sp. infection has been detected in eight cats included in the present study, which comprise 1.2% of the total number of cats analysed (644). The genus *Cytauxzoon* includes several species with *C. felis* being the most common in cats [35]. *Cytauxzoon felis* causes a generally highly fatal disease in domestic cats in the USA, with wild felids such as the bobcats (*Lynx rufus*) and pumas (*Puma concolor*) as the main reservoirs for this pathogen [35]. However, other species of *Cytauxzoon* infecting wild or domestic felids outside America have been described, including *C. manul*, molecularly characterized from a Pallas’ cat (*Otocolobus manul*) from Mongolia [36, 37], and genetically-close *Cytauxzoon* sp., reported in wild felids and domestic cats from Spain, France, Portugal and Italy [11, 22, 23, 25, 27, 38–41]. Information on the epidemiology and clinical manifestations of infection of domestic cats by other species distinct from *C. felis* is still scarce.

The analysis of sequenced PCR products showed that *Cytauxzoon* sp. detected in cats from Spain in the current study are very close to the previous isolates “*Cytauxzoon* sp. wild cat isolate 03/04326” (KT223486.1) from Spain [38], “*Cytauxzoon* sp. cat/France 1/2008” (EU622908.1) from France [11], and “*C. manul”* (KU710344.1) from Portugal [25]. As previously suggested, these isolates may belong to the same species, but further studies are needed to clarify the relatedness of the *Cytauxzoon* spp. infecting felids outside the USA [23].

The prevalence of *Cytauxzoon* sp. infection detected by PCR reported herein (1.2%) is lower than the previously described in Trieste region (north-eastern Italy), where 23% of domestic cats were found to be infected by this agent [23]. As described for *C. felis*, wild felids can serve as reservoirs for this infection in the domestic cat. In Spain, *Cytauxzoon* sp. infects the Iberian lynx (*Lynx pardinus*) and wildcats (*F. s. silvestris*) [27, 38–40]. Due to the extremely low population size of the Iberian lynx, it has been suggested that most likely, the natural reservoir for *Cytauxzoon* sp. in the Iberian Peninsula is the more abundant wildcat [35]. Interestingly, recent studies have shown infection by *C. felis* in healthy domestic cats, supporting the idea that not only wild felids are competent reservoirs for this *Cytauxzoon* [42]. European domestic cats, especially those who roam freely, could be acting as reservoirs for the *Cytauxzoon* sp. present in these areas [23, 35].

In our study, cats living in rural areas had a higher prevalence for this infection, with no amplification of *Cytauxzoon* spp. DNA from cats living in urban areas. This finding could be related to a higher exposure to the currently unknown potential tick vector. In agreement with that, the majority of the cats with *Cytauxzoon* sp. infection in the present study had outdoor access (75%, 6/8). Another *Cytauxzoon* sp.-positive cat had no outdoor access, while information about outdoor access was unkown for the last cat positive for this agent. Similarly, Carli et al. [23] described in an epidemiological study carried out in colony and owned cats from Italy that *Cytauxzoon* sp. infection was significantly associated with being free-ranging when compared with owned cats from the same city, suggesting a higher risk of this infection in cats exposed to potential tick vectors and to wildlife reservoirs, with a poor nutritional and clinical status and without preventative treatment against ectoparasites.

Surprisingly, our study showed a higher prevalence of *Cytauxzoon* sp. infection in samples collected during the colder months of the year, with the majority of positive cats (6/8) included in the study between January and March. This finding could be related to a chronic infection, but the possibility of the infection taking place in winter months cannot be ruled out. On the contrary, cytauxzoonosis caused by *C. felis* in the USA has been described to show a seasonal incidence from spring to early autumn [43]. Further studies with a large number of *Cytauxzoon* sp.-infected animals are needed to clarify transmission routes and epidemiology aspects of this agent in Europe.

We did not find any association between the presence or absence of clinical signs and *Cytauxzoon* sp. infection in the cats of the study. The occurrence of a fatal disease in a cat from Portugal infected by *Cytauxzoon* sp. has recently been described [25], but subclinical infection was highly prevalent in another epidemiological study [23]. *Cytauxzoon felis* infection has been described to frequently cause anemia in the domestic cat [21, 44, 45] and Criado-Fornelio et al. [22] described the presence of low haematocrit and haemoglobin values in a Spanish *Cytauxzoon* sp.-infected cat that also showed an increased level of glucose, hepatic enzymes and serum albumin. Only one of the *Cytauxzoon* sp.-infected cat in our study that had haematological and biochemical results showed slight normocytic, normochromic anemia, lymphopenia, thrombocytopenia and hyperproteinemia. However, this cat was also seropositive for *B. henselae* and FIV-positive. In this sense, another *Cytauxzoon* sp.-positive cat in our study was FIV positive, with statistically significant association between both infections. No previous relation has previously been described between *Cytauxzoon* spp. and retroviral infections [23, 46].

Finally, it should be considered that our study showed a higher risk for positivity to hemoprotozoans in stray cats compared to client-owned cats. This warrants further studies to evaluate the possible reservoir role of free roaming cats for these and other vector-borne pathogens.

## Conclusions

In summary, our results indicate that cats from Madrid are infected with *Hepatozoon* spp. and *Cytauxzoon* sp., although with a low prevalence. A decrease in haematocrit value and an increase in creatinine value were associated with *Hepatozoon* spp. infection*. Cytauxzoon* sp. infection was more frequent in samples collected during winter months and in cats living in rural areas and was associated with a FIV-positive status. Infection with these agents did not show association with the clinical status or lifestyle (client-owned/stray) of the cats included in the study. Further studies are needed to clarify epidemiological and clinical aspects of these infections in Spain.
